# Aqua­(2,9-dimethyl-1,10-phenanthroline-κ^2^ *N*,*N*′)(formato-κ^2^ *O*,*O*′)(formato-κ*O*)cobalt(II) monohydrate

**DOI:** 10.1107/S1600536810050051

**Published:** 2010-12-04

**Authors:** Sheng-Liang Ni, Ping Xia, Feng Cao

**Affiliations:** a Department of Chemistry, Huzhou Teachers College, Huzhou Key Laboratory Base of Novel Functional Materials, Huzhou, Zhejiang 313000, People’s Republic of China

## Abstract

The asymmetric unit of the title compound, [Co(HCO_2_)_2_(C_14_H_12_N_2_)(H_2_O)]·H_2_O, contains a mononuclear complex mol­ecule hydrogen bonded to a lattice water mol­ecule. The Co^II^ cation is in a distorted octa­hedral coordination environment defined by the two N atoms of the 2,9-dimethyl-1,10-phenanthroline ligand and four O atoms. Two of these are from a chelating formate anion, one from a monodentate formate and the last from an aqua ligand. In the crystal, mol­ecules are connected by O—H⋯O hydrogen bonds, forming double chains along [100] with the 2,9-dimethyl-1,10-phenanthroline ligands pointing outwards from each chain. These chains are further linked into layers parallel to (011) by inter-chain π–π stacking inter­actions with centroid–centroid distances of 3.61 (1) Å.

## Related literature

For background to the formation and applications of supra­molecular metal complexes, see: Moulton & Zaworotko (2001 ▶); Aakeroy & Seddon (1993 ▶). For related structures, see: Cai *et al.* (2008 ▶); Chen *et al.* (2009 ▶).
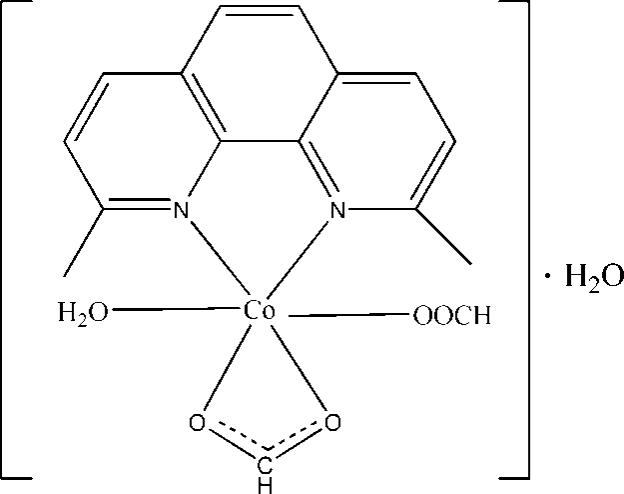


## Experimental

### 

#### Crystal data

[Co(HCO_2_)_2_(C_14_H_12_N_2_)(H_2_O)]·H_2_O
*M* *_r_* = 393.25
Triclinic, 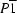

*a* = 7.4220 (15) Å
*b* = 10.441 (2) Å
*c* = 11.419 (2) Å
α = 82.92 (3)°
β = 81.62 (3)°
γ = 76.01 (3)°
*V* = 845.9 (3) Å^3^
*Z* = 2
Mo *K*α radiation
μ = 1.05 mm^−1^
*T* = 295 K
0.16 × 0.10 × 0.08 mm

#### Data collection

Bruker P4 diffractometer
Absorption correction: ψ scan (*XSCANS*; Siemens, 1996 ▶) *T*_min_ = 0.880, *T*_max_ = 0.912
4797 measured reflections
3897 independent reflections
3362 reflections with *I* > 2σ(*I*)
*R*_int_ = 0.017
3 standard reflections every 97 reflections  intensity decay: none

#### Refinement

*R*[*F* ^2^ > 2σ(*F* ^2^)] = 0.035
*wR*(*F* ^2^) = 0.098
*S* = 1.03
3897 reflections
227 parameters
6 restraints
H-atom parameters constrained
Δρ_max_ = 0.37 e Å^−3^
Δρ_min_ = −0.34 e Å^−3^

### 

Data collection: *XSCANS* (Siemens, 1996 ▶); cell refinement: *XSCANS*; data reduction: *XSCANS*; program(s) used to solve structure: *SHELXS97* (Sheldrick, 2008 ▶); program(s) used to refine structure: *SHELXL97* (Sheldrick, 2008 ▶); molecular graphics: *SHELXTL* (Sheldrick, 2008 ▶) and *DIAMOND* (Brandenburg, 2006 ▶); software used to prepare material for publication: *SHELXL97*.

## Supplementary Material

Crystal structure: contains datablocks global, I. DOI: 10.1107/S1600536810050051/sj5068sup1.cif

Structure factors: contains datablocks I. DOI: 10.1107/S1600536810050051/sj5068Isup2.hkl

Additional supplementary materials:  crystallographic information; 3D view; checkCIF report

## Figures and Tables

**Table 1:** Selected bond lengths (Å)

Co—O4	2.0552 (16)
Co—O1	2.0710 (17)
Co—N1	2.1194 (18)
Co—N2	2.1430 (16)
Co—O3	2.1622 (18)
Co—O2	2.2024 (17)

**Table 2:** Hydrogen-bond geometry (Å, °)

*D*—H⋯*A*	*D*—H	H⋯*A*	*D*⋯*A*	*D*—H⋯*A*
O1—H1*B*⋯O5^i^	0.85	1.84	2.688	173
O1—H1*C*⋯O6^ii^	0.84	1.98	2.774	156
O6—H6*B*⋯O3	0.85	1.96	2.809	176
O6—H6*C*⋯O4^iii^	0.85	2.33	3.098	151

Symmetry codes: (i) 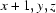; (ii) 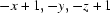; (iii) 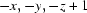.

**Fig. 1.**
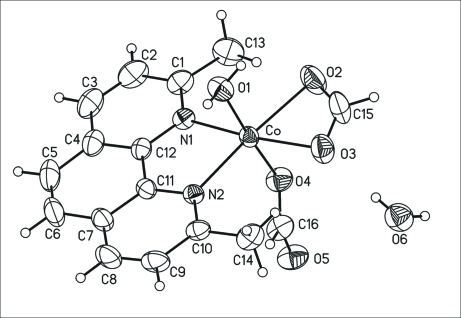
ORTEP view of the title compound. The displacement ellipsoids are drawn at 45% probability level.

**Fig. 2.**
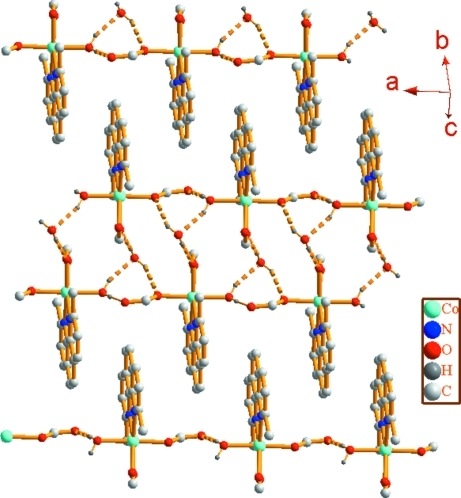
The two-dimensional layer structure of the title compound linked through hydrogen bonds and π–π stacking interactions.

## supplementary crystallographic information

### Comment

In the past decade, a variety of supramolecular architectures based on non–covalent intermolecular interactions such as hydrogen bonding, van der Walls forces and π–π interactions have been achieved by using transition metal centers and organic ligands due to their possible intriguing structural topologies and potential applications in optics, catalysis, ion exchange, gas storage, and the molecular–based magnetic materials (Aakeroy & Seddon, 1993). Carboxylate ligands have been actively utilized as construction units to obtain many supramolecular complexes (Moulton & Zaworotko,  2001) Herein, we are interested in self–assemblies of Co^II^ ions and 2,9-dimethyl-1,10-phenanthroline with formic acid, leading to the successful preparation of the complex [Co(H_2_O)(C_14_H_12_N_2_)(HCO_2_)_2_].H_2_O.

The asymmetric unit of the title compound consists of one Co^II^ ion, a H_2_O molecule, a 2,9-dimethyl-1,10-phenanthroline molecule, one O,*O*'–chelated formate anion and another coordinated formate anion, and one lattice H_2_O molecule (Fig. 1). Each Co^II^ atom is coordinated two N atoms from one 2,9-dimethyl-1,10-phenanthroline, three O atoms from two formate anions and one aqua ligand to complete a distorted octahedral CoN_2_O_4_ chromophore. The Co–N/O distances of 2.055 (2)–2.143 (2) Å, Table 1, fall within the normal range (Cai *et al.*, 2008, Chen *et al.*, 2009), both the *cisoid* and *transoid* bond angles in the range 59.90 (8)–112.35 (7) Å and 167.31 (7)–176.05 (6) Å, respectively, indicating that the octahedral CoN_2_O_4_ geometry is a highly distorted one. For the two formate anions, the angle (O2–C15–O3, 122.6 (3)°) in the chelated formate is smaller than that in the anion that binds in a monodentate fashion (O4–C16–O5, 128.8 (2)°). The 2,9-dimethyl-1,10-phenanthroline ligand is almost coplanar with a mean square deviation 0.0163 Å.  The O6 solvent water molecule is not coordinated to Co atom with the distance between the cobalt and water oxygen atoms of 4.800 (2) Å.  However it is linked to the complex molecule in the asymmetric unit by an O6—H6B—O3 hydrogen bond.

The molecules are connected by (O1–H1B···O5^#1^, O1–H1C···O6^#2^ and O6–H6C···O4^#3^) hydrogen bonds, Table 1, to form one-dimensional double chains along [100] with 2,9-dimethyl-1,10-phenanthroline orientating outwards.  The resulting chains are further linked into two-dimensional layers parallel to (011) by interchain π–π stacking interactions with centroid – centroid distances 3.61 (1) Å), Fig. 2.

### Experimental

Dropwise addition of 2.0 ml of 1.0 *M* aqueous Na_2_CO_3_ to a stirred aqueous solution of CoCl_2_.6H_2_O (0.2380 g, 1.0 mmol) in 5.0 ml H_2_O produced a pink precipitate, Co(OH)_2–2x_ (CO_3_)*_x_*.yH_2_O, which was centrifuged and washed with water until no Cl^-^ anions were detected in the supernatant. The precipitate was added to a stirred aqueous methanolic solution of 2,9-dimethyl-1,10-phenanthroline in 30 ml CH_3_OH–H_2_O (1/1 *v*/*v*). 1.77 ml of 1.0 *M* aqueous formic acid was added dropwise and stirred continuously until the pink precipitate dissolved. The pink solution (pH = 4.06) was allowed to stand at room temperature. Slow evaporation over several days afforded pink block shaped crystals. Yield:45% based on the initial CoCl_2_.6H_2_O input.

### Refinement

All H-atoms bound to C were positioned geometrically and refined using a riding model with  d(C-H) = 0.93Å,  U_iso_=1.2U_eq_ (C) for aromatic 0.93Å,  U_iso_ = 1.2U_eq_ (C) for CH and 0.96Å,  U_iso_ = 1.5U_eq_ (C) for CH_3_ atoms. H atoms attached to O atoms were found in a difference Fourier synthesis and were refined using a riding model, with the O–H distances fixed as initially found and with *U*_iso_(H) values set at 1.5 *U*eq(O).

### Crystal data

**Table:**

[Co(HCO_2_)_2_(C_14_H_12_N_2_)(H_2_O)]·H_2_O	*Z* = 2
*M**_r_* = 393.25	*F*(000) = 406
Triclinic, *P*1	*D*_x_ = 1.544 Mg m^−^^3^
Hall symbol: -P 1	Mo *K*α radiation, λ = 0.71073 Å
*a* = 7.4220 (15) Å	Cell parameters from 25 reflections
*b* = 10.441 (2) Å	θ = 5.0–12.5°
*c* = 11.419 (2) Å	µ = 1.05 mm^−^^1^
α = 82.92 (3)°	*T* = 295 K
β = 81.62 (3)°	Block, pink
γ = 76.01 (3)°	0.16 × 0.10 × 0.08 mm
*V* = 845.9 (3) Å^3^	

### Data collection

**Table:**

Bruker P4 diffractometer	3362 reflections with *I* > 2σ(*I*)
Radiation source: fine-focus sealed tube	*R*_int_ = 0.017
graphite	θ_max_ = 27.5°, θ_min_ = 1.8°
θ/2θ scans	*h* = −1→9
Absorption correction: ψ scan (*XSCANS*; Siemens, 1996)	*k* = −13→13
*T*_min_ = 0.880, *T*_max_ = 0.912	*l* = −14→14
4797 measured reflections	3 standard reflections every 97 reflections
3897 independent reflections	intensity decay: none

### Refinement

**Table:**

Refinement on *F*^2^	Secondary atom site location: difference Fourier map
Least-squares matrix: full	Hydrogen site location: inferred from neighbouring sites
*R*[*F*^2^ > 2σ(*F*^2^)] = 0.035	H-atom parameters constrained
*wR*(*F*^2^) = 0.098	*w* = 1/[σ^2^(*F*_o_^2^) + (0.0543*P*)^2^ + 0.2279*P*] where *P* = (*F*_o_^2^ + 2*F*_c_^2^)/3
*S* = 1.03	(Δ/σ)_max_ < 0.001
3897 reflections	Δρ_max_ = 0.37 e Å^−^^3^
227 parameters	Δρ_min_ = −0.34 e Å^−^^3^
6 restraints	Extinction correction: *SHELXL97* (Sheldrick, 2008), Fc^*^=kFc[1+0.001xFc^2^λ^3^/sin(2θ)]^-1/4^
Primary atom site location: structure-invariant direct methods	Extinction coefficient: 0.002468

### Special details

**Table:**

Geometry. All e.s.d.'s (except the e.s.d. in the dihedral angle between two l.s. planes) are estimated using the full covariance matrix. The cell e.s.d.'s are taken into account individually in the estimation of e.s.d.'s in distances, angles and torsion angles; correlations between e.s.d.'s in cell parameters are only used when they are defined by crystal symmetry. An approximate (isotropic) treatment of cell e.s.d.'s is used for estimating e.s.d.'s involving l.s. planes.
Refinement. Refinement of *F*^2^ against ALL reflections. The weighted *R*-factor *wR* and goodness of fit *S* are based on *F*^2^, conventional *R*-factors *R* are based on *F*, with *F* set to zero for negative *F*^2^. The threshold expression of *F*^2^ > σ(*F*^2^) is used only for calculating *R*-factors(gt) *etc*. and is not relevant to the choice of reflections for refinement. *R*-factors based on *F*^2^ are statistically about twice as large as those based on *F*, and *R*- factors based on ALL data will be even larger.

### Fractional atomic coordinates and isotropic or equivalent isotropic displacement parameters (Å^2^)

**Table:**

	*x*	*y*	*z*	*U*_iso_*/*U*_eq_	
Co	0.27534 (4)	0.17941 (2)	0.27175 (2)	0.03146 (11)	
N1	0.2555 (2)	0.25976 (17)	0.09313 (14)	0.0331 (3)	
N2	0.2662 (2)	0.38360 (15)	0.28646 (14)	0.0305 (3)	
O1	0.5639 (2)	0.15052 (15)	0.24594 (14)	0.0452 (4)	
H1B	0.6115	0.2032	0.2767	0.068*	
H1C	0.6274	0.0738	0.2635	0.068*	
O2	0.3174 (3)	−0.03795 (17)	0.29174 (18)	0.0637 (5)	
O3	0.2798 (3)	0.07298 (17)	0.44656 (15)	0.0551 (4)	
O4	−0.0089 (2)	0.19581 (17)	0.29267 (15)	0.0474 (4)	
O5	−0.2919 (2)	0.30672 (19)	0.35861 (16)	0.0539 (4)	
O6	0.1515 (3)	0.06203 (18)	0.69063 (17)	0.0593 (5)	
H6B	0.1849	0.0670	0.6158	0.089*	
H6C	0.0747	0.0117	0.7018	0.089*	
C1	0.2454 (3)	0.1974 (2)	−0.0004 (2)	0.0452 (5)	
C2	0.2397 (4)	0.2645 (3)	−0.1146 (2)	0.0577 (7)	
H2A	0.2335	0.2194	−0.1787	0.069*	
C3	0.2432 (4)	0.3945 (3)	−0.1321 (2)	0.0576 (7)	
H3A	0.2416	0.4381	−0.2081	0.069*	
C4	0.2491 (3)	0.4633 (2)	−0.03500 (19)	0.0448 (5)	
C5	0.2526 (4)	0.6003 (3)	−0.0464 (2)	0.0587 (7)	
H5A	0.2480	0.6482	−0.1206	0.070*	
C6	0.2625 (4)	0.6611 (2)	0.0489 (3)	0.0575 (7)	
H6A	0.2655	0.7503	0.0395	0.069*	
C7	0.2686 (3)	0.5909 (2)	0.1641 (2)	0.0436 (5)	
C8	0.2817 (4)	0.6496 (2)	0.2655 (2)	0.0543 (6)	
H8A	0.2870	0.7383	0.2597	0.065*	
C9	0.2868 (4)	0.5763 (2)	0.3717 (2)	0.0504 (6)	
H9A	0.2964	0.6150	0.4390	0.060*	
C10	0.2776 (3)	0.4427 (2)	0.38166 (18)	0.0367 (4)	
C11	0.2631 (3)	0.45609 (18)	0.17911 (18)	0.0320 (4)	
C12	0.2558 (3)	0.39041 (19)	0.07673 (17)	0.0330 (4)	
C13	0.2345 (5)	0.0557 (3)	0.0209 (3)	0.0657 (8)	
H13A	0.2420	0.0262	0.1034	0.099*	
H13B	0.3364	0.0031	−0.0266	0.099*	
H13C	0.1181	0.0466	−0.0004	0.099*	
C14	0.2786 (4)	0.3641 (3)	0.5002 (2)	0.0500 (6)	
H14A	0.2716	0.2752	0.4914	0.075*	
H14B	0.1729	0.4044	0.5531	0.075*	
H14C	0.3918	0.3622	0.5324	0.075*	
C15	0.3054 (5)	−0.0333 (3)	0.4003 (3)	0.0628 (7)	
H15	0.3158	−0.1120	0.4494	0.075*	
C16	−0.1206 (3)	0.2801 (2)	0.3492 (2)	0.0467 (5)	
H16	−0.0681	0.3306	0.3905	0.056*	

### Atomic displacement parameters (Å^2^)

**Table:**

	*U*^11^	*U*^22^	*U*^33^	*U*^12^	*U*^13^	*U*^23^
Co	0.03515 (17)	0.02810 (15)	0.03361 (16)	−0.01129 (10)	−0.00462 (10)	−0.00368 (10)
N1	0.0329 (8)	0.0380 (9)	0.0320 (8)	−0.0120 (7)	−0.0051 (6)	−0.0082 (6)
N2	0.0298 (8)	0.0298 (8)	0.0343 (8)	−0.0098 (6)	−0.0029 (6)	−0.0078 (6)
O1	0.0391 (8)	0.0407 (8)	0.0572 (10)	−0.0071 (6)	−0.0124 (7)	−0.0062 (7)
O2	0.0943 (15)	0.0355 (9)	0.0658 (12)	−0.0211 (9)	−0.0134 (11)	−0.0051 (8)
O3	0.0761 (12)	0.0468 (9)	0.0447 (9)	−0.0196 (9)	−0.0124 (8)	0.0042 (7)
O4	0.0358 (8)	0.0539 (9)	0.0561 (10)	−0.0159 (7)	−0.0003 (7)	−0.0135 (8)
O5	0.0345 (8)	0.0684 (11)	0.0627 (11)	−0.0125 (8)	−0.0044 (7)	−0.0209 (9)
O6	0.0680 (12)	0.0556 (10)	0.0575 (11)	−0.0154 (9)	−0.0121 (9)	−0.0099 (8)
C1	0.0466 (12)	0.0566 (14)	0.0382 (11)	−0.0176 (10)	−0.0055 (9)	−0.0143 (10)
C2	0.0650 (16)	0.0796 (19)	0.0350 (12)	−0.0207 (14)	−0.0096 (11)	−0.0173 (12)
C3	0.0606 (16)	0.0793 (19)	0.0319 (11)	−0.0174 (14)	−0.0071 (11)	0.0033 (11)
C4	0.0424 (12)	0.0518 (13)	0.0381 (11)	−0.0114 (10)	−0.0052 (9)	0.0064 (9)
C5	0.0621 (16)	0.0559 (15)	0.0535 (14)	−0.0178 (12)	−0.0079 (12)	0.0222 (12)
C6	0.0622 (16)	0.0355 (12)	0.0705 (17)	−0.0141 (11)	−0.0053 (13)	0.0146 (11)
C7	0.0420 (12)	0.0304 (10)	0.0580 (14)	−0.0110 (9)	−0.0009 (10)	−0.0030 (9)
C8	0.0588 (15)	0.0331 (11)	0.0744 (18)	−0.0166 (10)	0.0014 (13)	−0.0161 (11)
C9	0.0512 (13)	0.0456 (12)	0.0609 (15)	−0.0175 (10)	0.0029 (11)	−0.0282 (11)
C10	0.0333 (10)	0.0413 (11)	0.0392 (10)	−0.0118 (8)	−0.0012 (8)	−0.0153 (8)
C11	0.0279 (9)	0.0286 (9)	0.0398 (10)	−0.0072 (7)	−0.0037 (8)	−0.0028 (7)
C12	0.0291 (9)	0.0367 (10)	0.0332 (10)	−0.0089 (8)	−0.0042 (7)	−0.0003 (8)
C13	0.088 (2)	0.0612 (17)	0.0608 (16)	−0.0293 (15)	−0.0094 (15)	−0.0278 (13)
C14	0.0568 (14)	0.0630 (15)	0.0366 (11)	−0.0198 (12)	−0.0086 (10)	−0.0140 (10)
C15	0.086 (2)	0.0389 (13)	0.0647 (17)	−0.0232 (13)	−0.0157 (15)	0.0143 (12)
C16	0.0392 (12)	0.0604 (14)	0.0470 (12)	−0.0200 (11)	−0.0036 (10)	−0.0151 (11)

### Geometric parameters (Å, °)

**Table:**

Co—O4	2.0552 (16)	C3—H3A	0.9300
Co—O1	2.0710 (17)	C4—C12	1.404 (3)
Co—N1	2.1194 (18)	C4—C5	1.426 (4)
Co—N2	2.1430 (16)	C5—C6	1.345 (4)
Co—O3	2.1622 (18)	C5—H5A	0.9300
Co—O2	2.2024 (17)	C6—C7	1.426 (4)
Co—C15	2.488 (3)	C6—H6A	0.9300
N1—C1	1.337 (3)	C7—C8	1.401 (3)
N1—C12	1.354 (3)	C7—C11	1.406 (3)
N2—C10	1.337 (2)	C8—C9	1.353 (4)
N2—C11	1.359 (3)	C8—H8A	0.9300
O1—H1B	0.8536	C9—C10	1.403 (3)
O1—H1C	0.8425	C9—H9A	0.9300
O2—C15	1.236 (3)	C10—C14	1.494 (3)
O3—C15	1.249 (3)	C11—C12	1.440 (3)
O4—C16	1.229 (3)	C13—H13A	0.9600
O5—C16	1.225 (3)	C13—H13B	0.9600
O6—H6B	0.8529	C13—H13C	0.9600
O6—H6C	0.8512	C14—H14A	0.9600
C1—C2	1.404 (4)	C14—H14B	0.9600
C1—C13	1.489 (4)	C14—H14C	0.9600
C2—C3	1.353 (4)	C15—H15	0.9300
C2—H2A	0.9300	C16—H16	0.9300
C3—C4	1.405 (4)		
			
O4—Co—O1	176.05 (6)	C6—C5—C4	120.8 (2)
O4—Co—N1	88.20 (7)	C6—C5—H5A	119.6
O1—Co—N1	91.40 (7)	C4—C5—H5A	119.6
O4—Co—N2	96.90 (7)	C5—C6—C7	121.1 (2)
O1—Co—N2	86.88 (7)	C5—C6—H6A	119.5
N1—Co—N2	79.03 (7)	C7—C6—H6A	119.5
O4—Co—O3	87.65 (8)	C8—C7—C11	117.2 (2)
O1—Co—O3	92.21 (8)	C8—C7—C6	123.0 (2)
N1—Co—O3	171.25 (6)	C11—C7—C6	119.8 (2)
N2—Co—O3	109.13 (7)	C9—C8—C7	119.5 (2)
O4—Co—O2	89.29 (8)	C9—C8—H8A	120.3
O1—Co—O2	87.23 (8)	C7—C8—H8A	120.3
N1—Co—O2	112.35 (7)	C8—C9—C10	120.8 (2)
N2—Co—O2	167.32 (7)	C8—C9—H9A	119.6
O3—Co—O2	59.90 (8)	C10—C9—H9A	119.6
O4—Co—C15	88.17 (9)	N2—C10—C9	121.1 (2)
O1—Co—C15	89.74 (9)	N2—C10—C14	118.73 (18)
N1—Co—C15	141.97 (9)	C9—C10—C14	120.1 (2)
N2—Co—C15	138.97 (9)	N2—C11—C7	122.92 (19)
O3—Co—C15	30.14 (9)	N2—C11—C12	117.94 (16)
O2—Co—C15	29.76 (8)	C7—C11—C12	119.14 (19)
C1—N1—C12	118.95 (18)	N1—C12—C4	122.82 (19)
C1—N1—Co	128.10 (15)	N1—C12—C11	117.96 (17)
C12—N1—Co	112.95 (13)	C4—C12—C11	119.22 (19)
C10—N2—C11	118.44 (17)	C1—C13—H13A	109.5
C10—N2—Co	129.34 (14)	C1—C13—H13B	109.5
C11—N2—Co	111.99 (12)	H13A—C13—H13B	109.5
Co—O1—H1B	117.2	C1—C13—H13C	109.5
Co—O1—H1C	117.6	H13A—C13—H13C	109.5
H1B—O1—H1C	105.8	H13B—C13—H13C	109.5
C15—O2—Co	88.00 (15)	C10—C14—H14A	109.5
C15—O3—Co	89.50 (16)	C10—C14—H14B	109.5
C16—O4—Co	123.21 (15)	H14A—C14—H14B	109.5
H6B—O6—H6C	104.7	C10—C14—H14C	109.5
N1—C1—C2	121.0 (2)	H14A—C14—H14C	109.5
N1—C1—C13	118.0 (2)	H14B—C14—H14C	109.5
C2—C1—C13	121.0 (2)	O2—C15—O3	122.6 (2)
C3—C2—C1	120.4 (2)	O2—C15—Co	62.23 (13)
C3—C2—H2A	119.8	O3—C15—Co	60.36 (13)
C1—C2—H2A	119.8	O2—C15—H15	118.7
C2—C3—C4	119.8 (2)	O3—C15—H15	118.7
C2—C3—H3A	120.1	Co—C15—H15	179.0
C4—C3—H3A	120.1	O5—C16—O4	128.8 (2)
C12—C4—C3	117.0 (2)	O5—C16—H16	115.6
C12—C4—C5	120.0 (2)	O4—C16—H16	115.6
C3—C4—C5	123.0 (2)		

### Hydrogen-bond geometry (Å, °)

**Table:**

*D*—H···*A*	*D*—H	H···*A*	*D*···*A*	*D*—H···*A*
O1—H1B···O5^i^	0.85	1.84	2.688	173
O1—H1C···O6^ii^	0.84	1.98	2.774	156
O6—H6B···O3	0.85	1.96	2.809	176
O6—H6C···O4^iii^	0.85	2.33	3.098	151

Symmetry codes: (i) *x*+1, *y*, *z*; (ii) −*x*+1, −*y*, −*z*+1;  (iii) −*x*, −*y*, −*z*+1.
